# Supplementary material for: Genome-Wide Association Studies on the Kernel Row Number in a Multi-Parent Maize Population
Source: Int J Mol Sci. 2024 Mar 16;25(6):3377. doi: 10.3390/ijms25063377 (PMC10970222; doi:10.3390/ijms25063377)
Supplement: Supplementary file 1 [file ijms-25-03377-s001.zip › FIG S1.pdf]

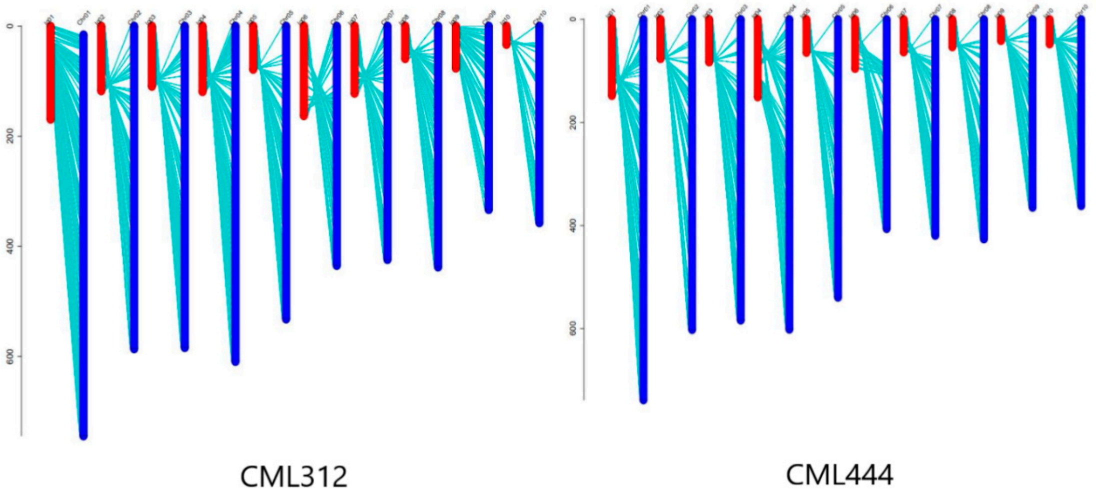

Figure S1: Co-linearity analysis between genetic and physical maps (Red represents 18 linkage groups, and blue represents chromosomes, i.e., physical maps.)
