# Supplementary material for: Genome-Wide Association Studies on the Kernel Row Number in a Multi-Parent Maize Population
Source: Int J Mol Sci. 2024 Mar 16;25(6):3377. doi: 10.3390/ijms25063377 (PMC10970222; doi:10.3390/ijms25063377)
Supplement: Supplementary file 1 [file ijms-25-03377-s001.zip › Supplemental_Tables_1_2.pdf]

Supplementary Table S1-2

Table S1: Significant SNPs obtained through GWAS†

Loc	num_snp	gene_num	peakPOS	Chr	Bin	PVE（%）	*P*(-log10)
21YS	1	2	242858336	2	2.24	17.74	5.98
	1	0	180296831	8	8.18	6.34	5.08
22YS	3	3	126528998	10	10.12	14.88	5.64
19BS	1	0	135828991	6	6.13	3.24	5.15
19DH	1	0	120370778	5	5.12	8.83	5.05
	5	2	177304649	7	7.17	6.40	6.03
21JH	1	31	76395713	1	1.07	9.09	5.16
	1		83163645	1	1.08	21.01	6.40
	1		198129347	1	1.19	7.02	6.01
	1		199106836	1	1.19	6.14	5.89
	1		199222030	1	1.19	8.42	6.64
	1		199314176	1	1.19	6.49	5.63
	1		199335283	1	1.19	4.73	5.18
	3		201355723	1	1.20	5.57	5.70
	1		254646097	1	1.25	14.18	6.05
	2		257676626	1	1.25	5.69	5.58
	1		306464753	1	1.30	8.58	5.56
	1		42354101	2	2.04	5.88	5.65
	1		104677372	2	2.10	9.92	5.79
	1		192141542	2	2.19	12.01	5.79
	1		195713294	2	2.19	6.56	5.36
	1		214756894	2	2.21	12.53	6.10
	3		215468689	2	2.21	14.31	6.12
	1		24283034	3	3.02	13.44	5.29
	1		135283055	4	4.13	6.70	6.13
	1		187159704	4	4.18	9.96	6.37
	4		46725146	5	5.04	10.57	8.39
	2		138554600	5	5.13	8.40	5.42
	1		150393177	5	5.15	10.64	5.98
	1		30305673	6	6.03	8.89	6.31
	1		71741394	6	6.07	7.79	5.82
	2		71920788	6	6.07	6.33	6.24
	1		76471294	6	6.07	8.70	5.98
	2		84033777	8	8.08	9.61	8.61
	1		146316294	8	8.14	5.61	5.95
	1		68447065	10	10.06	11.15	5.00
SUM	53	38					

†Loc: Refers to the Environment or Location where the association was detected.
Chr: Indicates the Chromosome on which the significant SNP is located.
PVE: Represents the Proportion of Phenotypic Variance Explained by the associated SNP.
P: Represents the p-value.

Table S2: Summary of Genetic Linkage Groups of two RIL populations†

population	Chr	Marker number	Length (cM)	inter marker	Max−gap
	lg01	180	169	0.94	5.95
	lg02	93	117.35	1.26	9.08
	lg03	117	109.28	0.93	10.05
	lg04	131	118.91	0.91	4.72
	lg05	85	78.41	0.92	9.56
Sub-pop3	lg06	91	162.62	1.79	17.5
	lg07	96	121.87	1.27	12.12
	lg08	84	58.93	0.7	5.54
	lg09	66	76.24	1.16	13.78
	lg10	38	33.23	0.87	4.33
	**Total**	**981**	**1045.83**	**1.07**	**17.5**
	lg01	144	148.39	1.03	17.75
	lg02	78	76.86	0.99	8.47
	lg03	89	83.28	0.94	11.78
	lg04	109	151.08	1.39	28.89
	lg05	80	64.76	0.81	6.6
Sub-pop4	lg06	61	95.89	1.57	12.68
	lg07	80	63.94	0.8	8.47
	lg08	54	53.92	1	9.26
	lg09	56	41.88	0.75	6.24
	lg10	70	48.27	0.69	3.56
	**Total**	**821**	**828.27**	**1.01**	**28.89**

†lg1,lg2,…lg10 represent chromosome 1,2, …10, respectively. Marker-number: indicates the number of SNP markers. Length: The total genetic distance of each chromosome (cM); Average_length: average genetic distance (cM). Max-gap: refers to the maximum allowable gap or distance between adjacent genetic markers (usually microsatellites or SNPs) along a chromosome.
