# Supplementary material for: Genome-Wide Association Studies on the Kernel Row Number in a Multi-Parent Maize Population
Source: Int J Mol Sci. 2024 Mar 16;25(6):3377. doi: 10.3390/ijms25063377 (PMC10970222; doi:10.3390/ijms25063377)
Supplement: Supplementary file 1 [file ijms-25-03377-s001.zip › Supplemental_Tables_4.pdf]

Supplementary Table S4

Table S4 Consistently detected QTLs in six environments and BLUP in two sub-populations (Pop)†

Pop	Loc	Marker	Chr	Position(cM)	LOD
Sub-pop3	19BS	mk563	5	47.81	2.813415
		mk562	5	48.17	2.947647
		mk907	9	29.91	4.435625
		mk908	9	30.24	4.369219
		mk908	9	31.24	3.700967
		mk909	9	31.25	3.688945
		mk912	9	33.97	3.807416
	19DH	mk90	1	82.76	5.238167
		mk91	1	83.47	5.499796
		mk92	1	84.14	5.589958
		mk93	1	84.48	5.637543
		mk93	1	85.48	6.282973
		mk94	1	85.84	6.296636
		mk95	1	86.17	6.29901
		mk96	1	86.84	5.813353
		mk97	1	87.18	5.705053
		mk111	1	95.58	5.550556
		mk93	1	85.48	3.051815
		mk94	1	85.84	3.143676
		mk95	1	86.17	3.143905
		mk96	1	86.84	2.958989
		mk97	1	87.18	2.804033
		mk111	1	95.58	2.64608
		mk563	5	47.81	2.952361
		mk562	5	48.17	2.919248
		mk907	9	29.91	2.874622
		mk908	9	30.24	2.715203
		mk908	9	31.24	2.565146
		mk909	9	31.25	2.559051
		mk912	9	33.97	3.807416
	22YS	mk401	4	113.79	4.070378
		mk400	4	114.22	4.138289
		mk399	4	114.89	3.906295
		mk398	4	115.22	3.905721
		mk398	4	116.22	3.871544
		mk397	4	116.24	3.860575

		mk396	4	116.57	3.187334
		mk401	4	113.79	2.866335
		mk400	4	114.22	3.016834
		mk399	4	114.89	2.750404
	BLUP	mk398	4	115.22	2.734766
		mk398	4	116.22	2.650455
		mk397	4	116.24	2.651615
		mk396	4	116.57	2.688204
	21JH	mk396	4	76.5	4.163719
Sub-pop4	19BS	mk225	2	72.39	2.635027
	21YS	mk396	4	76.5	2.71844

† BLUP, the best linear unbiased prediction; 21YS, Yanshan in 2021; 21JH, Jinghong in 2021; 22YS, Yanshan in 2022; 19BS, Baoshan in 2019; 19DH, Dehong in 2019. Sub-pop3 and Sub-pop4：CML312 and CML444 lines were used for developing two subpopulations by crossing with Ye107. The same marker name between different populations does not necessarily indicate the same QTL. However, the same marker name indicates the same QTL within the same population.
