# Supplementary material for: Genome-Wide Association Studies on the Kernel Row Number in a Multi-Parent Maize Population
Source: Int J Mol Sci. 2024 Mar 16;25(6):3377. doi: 10.3390/ijms25063377 (PMC10970222; doi:10.3390/ijms25063377)
Supplement: Supplementary file 1 [file ijms-25-03377-s001.zip › Supplemental_Tables_7.pdf]

Supplementary Table S7

Table S7 QTLs that overlapped with cloned panicle-related genes

The QTLs identified in this study				Genes related to ear development that previous studies have cloned					
Loc	Chr	Start	End	Gene-ID	Chr	start	end	Function	reference
Sub-pop3_19DH	1	139616149	252952675	GRMZM2G011357	1	239,608,944	239,612,267	TF	Colasanti et al.
				GRMZM2G081554	1	241,218,603	241,226,854	Kaurene synth. (GA synthesis)	Bensen et al., 1995
				Zm00001d032961	1	244,858,795	244,867,417	ent-copy diphosphate synthase	Dellaporta et al 1994 Bensen et al. 1995
				Zm00001d031068	1	175,807,851	175,809,432	serine-threonine protein kinase	McSteen et al. 2001 McSteen et al. 2007 Skirpan et al. 2008 Skirpan et al. 2009
				Zm00001d032922	1	243,201,405	243,204,865	indeterminate domain protein	Colasanti et al. 1998

				Zm00001d031459	1	190,964,811	190,978,369	RNA polymerase IV	Erhard et al. 2009
				Zm00001d031451	1	190,382,866	190,386,589	SBP-box transcription factor	Chuck et al. 2014
----------------------	----	------------	-------------	----------------------	--	----------	--------------	-----------------	------------------
Sub-pop3_21JH	4	75,627,569	151,339,536	GRMZM2G104925	4	133,662,510	133,664,998	LRR receptor-like protein	Taguchi-Shiobara et al., 2001
				Zm00001d051012	4	136,765,871	136,767,712	LRR protein kinase	Taguchi-Shiobara et al 2001 Bommert et al. 2013b
----------------------	-----	------------	-------------	----------------------	--	----------	--------------	-----------------	------------------
Sub-pop4_22YS	4	46,403,895	172,368,609	GRMZM2G104925	4	133,662,510	133,664,998	LRR receptor-like protein	Taguchi-Shiobara et al., 2001
				Zm00001d051012	4	136,765,871	136,767,712	LRR protein kinase	Taguchi-Shiobara et al 2001 Bommert et al 2013b
Sub-pop4_21YS	5	76615734	181154647	Zm00001d016075	5	142,503,737	142,509,129	Negatively regulating KRN	An YX et al 2022
