# Supplementary figures and images for: Genome-Wide Association Studies on the Kernel Row Number in a Multi-Parent Maize Population
Source: Int J Mol Sci. 2024 Mar 16;25(6):3377. doi: 10.3390/ijms25063377 (PMC10970222; doi:10.3390/ijms25063377)

a

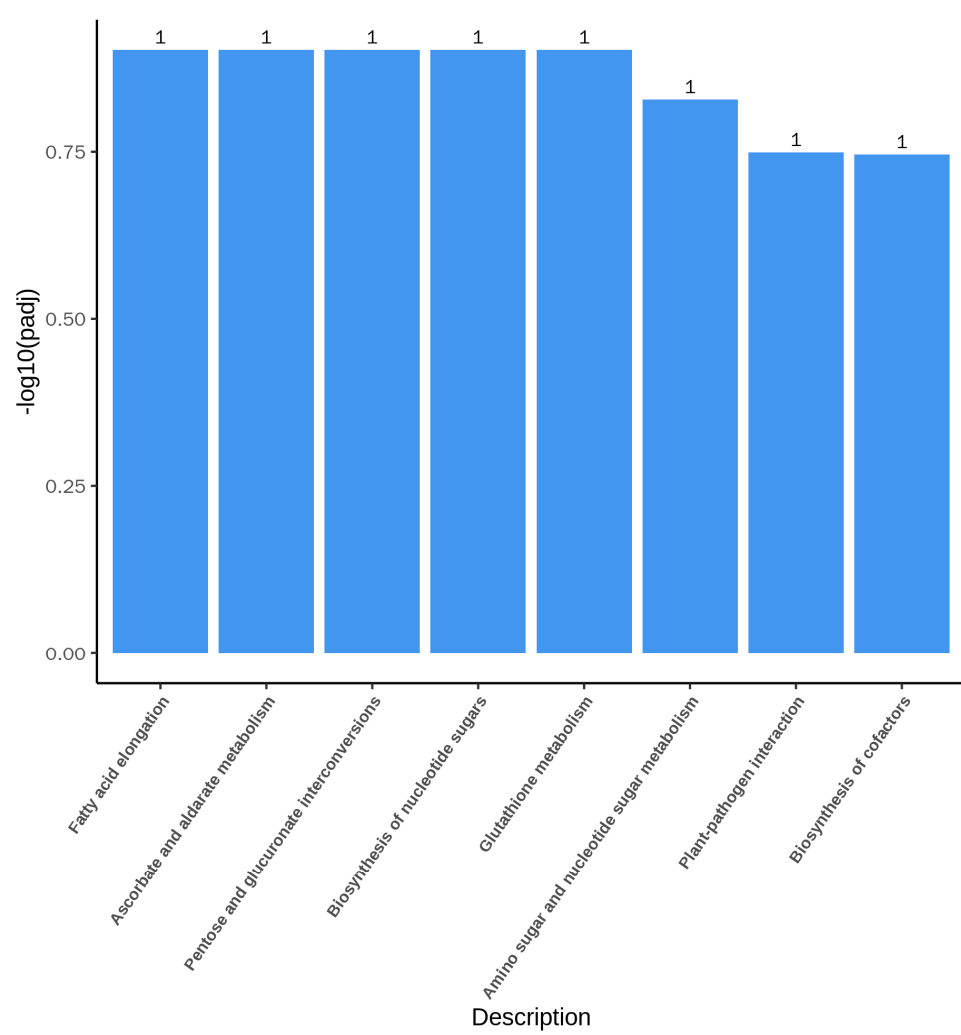

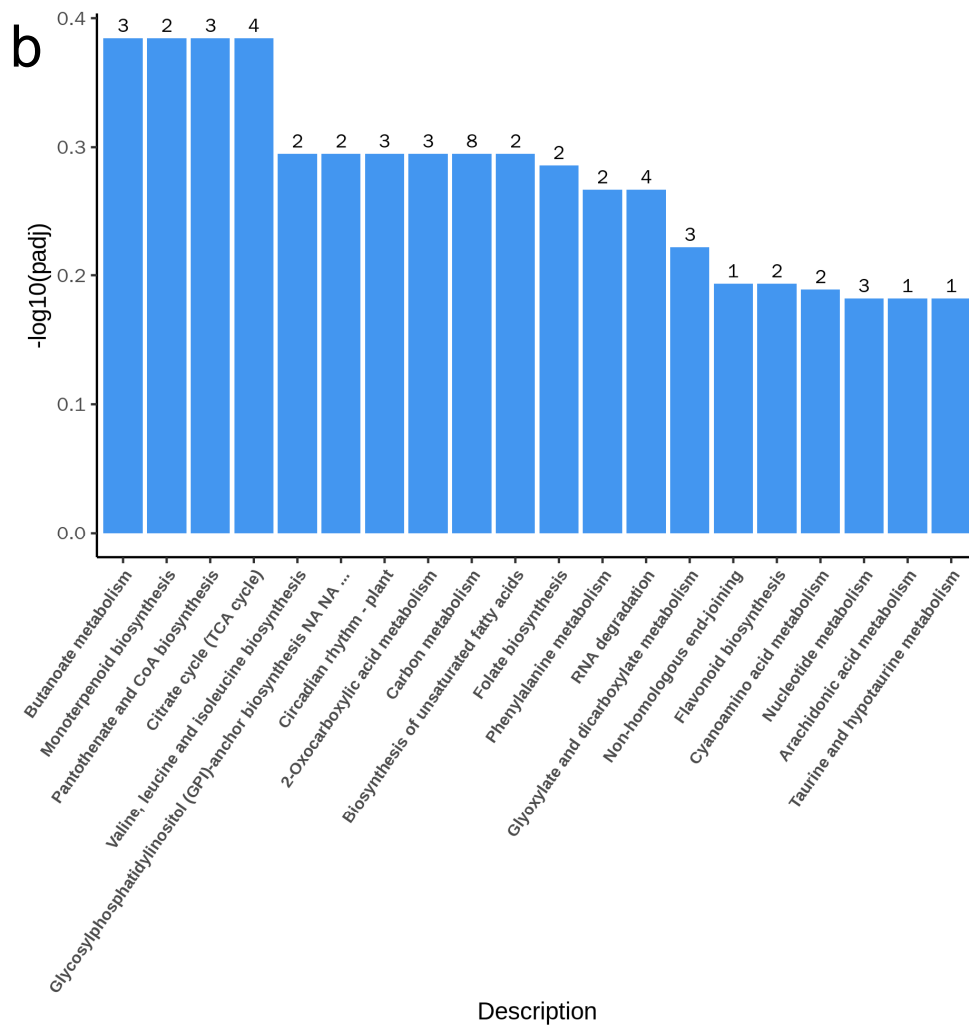

Supplement: Supplementary file 1 [file ijms-25-03377-s001.zip › FIG S2.pdf]
